# Dying well with reduced agency: a scoping review and thematic synthesis of the decision-making process in dementia, traumatic brain injury and frailty

**DOI:** 10.1186/s12910-016-0129-x

**Published:** 2016-07-27

**Authors:** Giles Birchley, Kerry Jones, Richard Huxtable, Jeremy Dixon, Jenny Kitzinger, Linda Clare

**Affiliations:** 1 Centre for Ethics in Medicine, University of Bristol, Bristol, UK; 2 Faculty of Health and Social Care, The Open University, Milton Keynes, UK; 3 Department of Social and Policy Sciences, University of Bath, Bath, UK; 4 Coma and Disorders of Consciousness Research Centre, Cardiff University, Cardiff, UK; 5 REACH: The Centre for Research in Ageing and Cognitive Health, University of Exeter, Exeter, UK

**Keywords:** End-of-life, Good Death, Mental capacity, Decision-making, Scoping review, Thematic synthesis, Frailty, Dementia, Traumatic brain injury

## Abstract

**Background:**

In most Anglophone nations, policy and law increasingly foster an autonomy-based model, raising issues for large numbers of people who fail to fit the paradigm, and indicating problems in translating practical and theoretical understandings of ‘good death’ to policy. Three exemplar populations are frail older people, people with dementia and people with severe traumatic brain injury. We hypothesise that these groups face some over-lapping challenges in securing good end-of-life care linked to their limited agency. To better understand these challenges, we conducted a scoping review and thematic synthesis.

**Methods:**

To capture a range of literature, we followed established scoping review methods. We then used thematic synthesis to describe the broad themes emerging from this literature.

**Results:**

Initial searches generated 22,375 references, and screening yielded 49, highly heterogeneous, studies that met inclusion criteria, encompassing 12 countries and a variety of settings. The thematic synthesis identified three themes: the first concerned the processes of end-of-life decision-making, highlighting the ambiguity of the dominant shared decision-making process, wherein decisions are determined by families or doctors, sometimes explicitly marginalising the antecedent decisions of patients. Despite this marginalisation, however, the patient does play a role both as a social presence and as an active agent, by whose actions the decisions of those with authority are influenced. The second theme examined the tension between predominant notions of a good death as ‘natural’ and the drive to medicalise death through the lens of the experiences and actions of those faced with the actuality of death. The final theme considered the concept of antecedent end-of-life decision-making (in all its forms), its influence on policy and decision-making, and some caveats that arise from the studies.

**Conclusions:**

Together these three themes indicate a number of directions for future research, which are likely to be applicable to other conditions that result in reduced agency. Above all, this review emphasises the need for new concepts and fresh approaches to end of life decision-making that address the needs of the growing population of frail older people, people with dementia and those with severe traumatic brain injury.

**Electronic supplementary material:**

The online version of this article (doi:10.1186/s12910-016-0129-x) contains supplementary material, which is available to authorized users.

## Background

### Introduction

Humans have pondered what constitutes a good death for millennia. From Plato’s account of Socrates’ dignified death in *Phaedo* to more contemporary investigations [1–3], a range of literature considers practical and theoretical understandings of what constitutes a ‘good death’ and a strong palliative care movement works actively to promote a vision of good end-of-life care. Yet the translation of this activity to policy remains problematic. In the United Kingdom (UK) and most Anglophone nations, policy and law increasingly foster an autonomy-based model, championing a consumer-focused conception of health provision and a central axiom of western moral philosophy. The UK government’s end of life care strategy [4] explicitly takes this path by primarily addressing good end of life care within the paradigm of cancer, where active, mentally competent patients with predictable patterns of decline seek a good death by exercising choices over their treatment and care. While the quality of every death is important, this strategy risks marginalising the large numbers of people who fail to fit the paradigm.

This includes three exemplar populations: *frail older* people, who face problems caused by multi-morbidity and extreme old age, people with *dementia*, who face a long term decline amid a lack of understanding of the terminal nature of their illness, and people with the most *severe traumatic brain injury*, who are often relatively young and, in the most severe cases face a lifetime of dependency. We hypothesise that these groups face challenges in securing good end-of-life care. Where statistical data is available (for traumatic brain injury it is not), these represent rising populations [5–7], suggesting that an examination of the problems they face is timely.^1^

What, then, might it mean for people in these three groups to ‘die well’? The Dying Well with Reduced Agency (DWRA) project seeks to answer this question by bringing together researchers from four UK Universities with a shared interest in end-of-life decision-making for those who have impediments to their decision-making ability. This paper gives details of a scoping review and thematic synthesis that searched for common issues in end-of-life care for the three exemplar conditions we focused upon.

### Dying with reduced agency

Not wishing to limit ourselves to the legally defined notions of ‘mental capacity’, we adopt the concept of ‘reduced agency’ to encompass the impediments facing the frail elderly, those with dementia, and those with severe traumatic brain injury. This captures the sense that autonomy must mean not only an ability to make a decision, but also an ability to make that decision happen.

Agency is the ability of a person or thing to act and bring about change [8]. If we shelve philosophical questions of causation and consider that people are agents who have free will, our level of agency reflects our ability to act and bring about change – in people, in things. Since reduced agency reflects a reduced ability to make a decision effective, it is distinguishable from reduced mental capacity, which concerns the ability to make a decision. Dying well with reduced agency puts this inability into the context of the types of things people may decide to do (or have done) in order to have a good death. Thus, for example, people may want to die at home, but be too frail to assert their wishes. Alternatively, they may, while in good health, decide to make an advance care plan that they do not want to be aggressively resuscitated if they have an accident, but be powerless to make anyone respect this at the critical time.

## Methods

### Search methodology

The literature search followed the methodology for a scoping study proposed by Arksey and O’Malley [9]. This specifies a search conducted with a broad research question, with restrictive parameters set once a sense of the entire literature is gained. The results are delimited using inclusion and exclusion criteria similar to systematic review, but also admit time limits by utilising strict deadlines for searching and inclusion. Data is then extracted in a format amenable to synthesis, and reporting, of findings.

Choice of methodology is defined by both the opportunities and constraints faced by the research team, and DWRA required a rapid assessment of distinct literatures in order to gain a broad understanding of the challenges and concepts described within them. The breadth of the focus meant that we did not (over-)specify a distinct research question, although we required a way to manage potentially vast amounts references generated by database searching, and wished to do so in a rigorous and transparent way. The fact that we were aiming for breadth rather than depth of review ruled out systematic review, as well as more novel forms that were suitable for swift assessment of precisely defined topics, such as rapid review [10]. While scoping review may be amenable to in-depth study of a specific area, Arksey and O’Malley [9] also indicate that scoping review may be employed in a range of other circumstances including assessing the topology a diverse range of literature, identifying research gaps and summarising research findings. These aims seemed most in keeping with what we were hoping to achieve.

### Data sources

The methodology informed a search protocol devised by the second author (KJ). Database searches were guided by the following research questions:

- What do we know about decision making at the end-of-life for people with extreme frailty in old age, severe dementia and severe traumatic brain injury? In particular;
  - ○ Where do decisions happen?
  - ○ Who makes decisions?
  - ○ How are decisions made?
- What are the differences and similarities in decision-making between these groups?

The following keywords were selected on the basis of these research questions and combined with Boolean operators to identify studies: End-of-life; dying; death; hospital; community; home; care home; long term care; dementia; traumatic brain injury; brain damage; vegetative state; disorder of consciousness; decision-making; accident and emergency; hospital; admissions; death. Keyword searches were performed of the following databases: Medline; PsychINFO; CINAHL; Cochrane database of systematic reviews; Assia; Ageinfo (1945–2015). Additionally, individual electronic searches were conducted on the websites of the *Journal of Housing for the Elderly*, *Journal of Social Work and Long Term Care* and the *British Journal of General Practice*.^2^

### Inclusion criteria

**Fig. 1:**
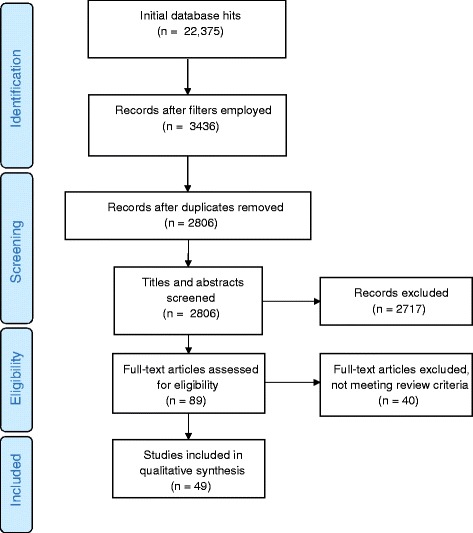
Flow diagram. Flow diagram of literature search and screen

Initial search results generated 22,375 references (see Fig. 1). These included: large volumes of clinical studies where the target groups and conditions were absent or constituted only small numbers of participants; legal, ethical and social scientific literature; editorials; and opinion pieces. While these potentially included items that were germane to the research question (for example literature on surrogate decision-making) we needed to reduce the literature to quantities that were manageable in the project timescale. We therefore decided to employ search filters to further focus the study upon empirical studies (reasoning these gave an indication of practice) that included a large proportion of the populations we sought to study:

- Addressed a population among whom frail older people, people with dementia and/or traumatic brain injury predominated (taken to be >75 % of participants);
- Reported original data (i.e. were empirical studies, not reviews or editorials/opinion pieces);
- Reported on decision-making related to end-of-life care in the community, care homes or hospital;
- Were written in English.

While dementia and traumatic brain injury are clinically well-defined, ‘frailty’ is a rich and contested label, and the screening process revealed that the literature contains many observational measures (which it was not the purpose of this search to review) that claim to detect frailty [11–15]. We therefore focused on papers that explicitly discussed the terms or concepts of ‘frailty’, ‘end-of-life decision-making’ and ‘older people’. Where this strategy was insufficient, frail old-age was taken to be aging with the capacity to make decisions but declining physical independence in daily activities.

### Data extraction

Papers were screened first by title, then by abstract. The full text of the papers returned was examined, with those that failed to meet the inclusion criteria being discarded. At each stage the inclusions and exclusions were examined independently by two authors (GB and KJ). A third reviewer (LC) made the final decision in cases where consensus could not be reached. Papers were not quality assessed as this does not form a part of the review method.

The full papers of studies that met the inclusion criteria and passed subsequent title and abstract reviews were retrieved and examined independently by the first two authors of this paper, GB and KJ. Each paper was reviewed and data – including sample size, study method, intervention characteristics, location, aims, results, and the agent of decision-making (e.g. the patient, relative and or clinician) – extracted using a pro-forma devised by KJ, and entered into a spreadsheet. In order to inform inductive conclusions, any additional study characteristics that appeared salient to the reviewers were also noted.

### Characteristics of reviewed studies

The 49 studies that met the inclusion criteria had different emphases. Sixteen were studies of frail older people, 25 were studies of dementia, eight of traumatic brain injury and. Most papers focused on the views, experiences, role or opinions of one or more participants in decisions pertinent to end-of-life care: 15 papers focused exclusively on family, friends, surrogates or other family carers; 14 focused only on clinicians; seven focused on both families and health care practitioners (including nurses, care assistants and doctors) or other professionals (such as social workers and care home managers); and five focused only on the person who was subject to the decision-making.

Settings of studies varied: 16 were conducted in community settings (including people’s homes, family doctor practices and day centres); 25 were undertaken in care homes (including nursing and group homes); 14 were conducted in hospitals; two were surveys conducted at international conferences. Unsurprisingly given the English language restriction, most studies were conducted in English-speaking societies, culturally sympathetic to individual autonomy [16]: 17 in the United States of America (USA); 12 in the UK; five in the Netherlands; three in Canada; two Hong-Kong; two Belgium; and one each in Australia, Germany, Israel, Italy, Japan and the Republic of Ireland; two were pan-European.

Study methods also varied. Eighteen were qualitative or mixed methods studies that relied on in-depth interview data from participants; 15 were observational studies (both prospective and retrospective) based on survey data, chart review or other forms of observational data; nine were epidemiological and used demographic data from large populations; five used interventions and/or quasi-experimental methods. The remaining two studies were case series. A summary of the studies is given in Additional file 1.

### Theme development

Since study methods and populations were heterogeneous, no effort was made to summarise quantitative data across the studies. A simple description of the data extracted was considered unlikely to inform the research questions; therefore, to maximise the value of the data, a process of qualitative synthesis was undertaken by the first author. Qualitative synthesis methods can be characterised by their aspiration to create a product that is more than the sum of its parts [17]. Thematic synthesis is a method of qualitative synthesis described by Thomas and Harden [18] that applies the qualitative method of thematic analysis (which itself arose from grounded theory) to literature review. Thomas and Harden describe three stages in such a synthesis. First, papers are coded line-by-line to pick out significant content. Second, topics within the codes are brought together under simple ‘descriptive themes’ that capture concepts common to groups of codes. Finally, ‘analytical themes’ are developed, consisting of researchers’ impressions and understandings of relationships between descriptive themes.

**Fig. 2:**
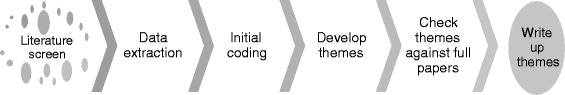
Thematic Synthesis. Diagrammatic representation of thematic synthesis

While utilising the method described, our method of deriving the initial codes differed (Fig. 2). With 49 papers, we had almost five times as much source material as Thomas and Harden [18], and limited time in which to extract it. Our solution was more explicitly iterative, since it involved frequent return to, and iteration between, the original sources. Since we were familiar with the papers from the process of data extraction, we used the secondary, extracted data (including the inductive notes which were made during the review) as initial codes while re-reading the papers to ensure that these codes accurately reflected the content, revising and refining the codes where necessary and adding new ones where gaps were apparent. Each paper was thus reread multiple times until the reviewer was content codes exhibited fealty to the original source. Through this approach, two categories were developed based upon the study characteristics. The first related to who the study methodology focused upon for information (e.g. families, doctors, patients) and the second upon what factor of the end of life process the study examined (e.g. limitations to treatment, decision-making processes). These categories were grouped into analytic themes which reflected reviewer impressions of ideas that occurred across multiple sources, and rich descriptions written. The descriptions were then checked against full papers for accuracy. Coding and theme development was primarily done by the first author (GB); however, at each stage the second author (KJ) checked the themes against the papers for accuracy, and themes were modified until consensus was reached. One paper from the search contained codes but did not exemplify the themes well enough to be included in the results below [19]. In total three descriptive themes arose from the literature. These themes focus upon the *processes of decision-making*, *limiting the intensity of medical treatment*, and *antecedent end-of-life decision-making*. The theme development process is illustrated in Additional files 2 and 3, with codes and themes arising from each paper given in Additional file 2, and descriptions of codes given in Additional file 3.

## Results

### Theme 1: decision-making processes

Decisions discussed in the papers concerned resuscitation, hospitalisation, surgery, ventilation, feeding via nasogastric tube or gastrostomy, and drug therapies such as antibiotics and sedation, as well as euthanasia in jurisdictions where this was legal. Such decision-making could happen in advance (via documentary or verbal evidence) or contemporaneously. In some cases advance and contemporaneous decisions conflicted.

The papers identified the distinctive roles of families and healthcare professionals in decision-making, but less often directly considered the patients themselves. Families often had different perspectives from healthcare professionals which, if unresolved, led to families expressing dissatisfaction with the dying process they witnessed. The universal approach to resolving such differences, as well as relieving the documented burdens on families of making decisions for dependent relatives, was shared decision-making, reflecting the current ethico-legal steer [20]. However, family opinion carried apparently different weight from one study to another, suggesting that decision-making was not being shared in a consistent manner. The patient’s own documented or verbal antecedent end-of-life decisions (by which we mean, among others, advance decisions and advance care plans, see below) might be considered, but whether these were followed depended on the outcome of medical assessment of the patient’s ‘best interests’, as well as the extent to which a family (if not agreeing with the antecedent decision) could assert their own wishes. Although rarely explicitly studied, close reading of many papers suggests that patients themselves may influence decision-making even when lacking capacity, and explicit study of this phenomena seems to represent a gap in the literature.

#### Who decides?

The papers presented clinical decision-making taking place either triadically, with the healthcare professional, patient and family member playing a role (e.g. [21]), or else dyadically, with the family and the healthcare professional making key decisions, albeit informed by their understanding of what the patient might have wanted (e.g. [22]). While it was rare to exclude *any* consideration of the family [23], dyadic conceptions often excluded the patient’s active contemporaneous involvement for practical reasons such as total patient incapacity [24, 25] or mortality [26]. But besides these practical reasons there was a clear, although unwritten, implication that the primary agents of decision-making were families and health care professionals, rather than the patients with reduced agency. Studies that directly involved patients with impaired capacity were rare [27, 28], while some implicitly conflated the experiences of families and patients [29].

#### Family and health care professional perspectives

Family perspectives can be discordant with those of clinical observers. Families of people with dementia were often disturbed by the symptoms experienced or exhibited by their relatives at the end of life, while clinical observers viewed them as consistent with a peaceful death [30–32]. Jox et al. [33] found almost a quarter of relatives of patients with traumatic brain injury disagreed with their doctor’s assessment of the patient’s level of consciousness. Families of patients in the acute stage of severe traumatic brain injury expected life-saving treatment either to be almost fully restorative or to fail completely, but few accepted that the likely result was a partial restoration of physical health alongside severe mental disability, in spite of receiving such information from clinicians [34]. Similar expectations were visible in the families of people with dementia, a long term degenerative condition [27, 35] where death by a catastrophic event (such as a cardiac arrest) was anticipated, rather than a slow and extended decline.

In some cases, healthcare professionals shared the family’s failure to acknowledge that dementia was a terminal condition. Sampson et al. [27] notes that many professionals in acute care were unaware that dementia was a terminal illness, and two papers [30, 36] observed that the death certificates of deceased patients who had advanced dementia often failed to note this diagnosis as a cause of death.

While doctors were in theory guided by the best interests of their patients, and empowered (in many jurisdictions) to make decisions on that basis, clinicians were aware that the variable opinions and philosophies within the healthcare team led to inconsistencies in practice, and this was considered to make any communication problems with families worse [37].

#### Impact on families

Dening et al. [38] noted that family carers of people with dementia felt the toll of caring and often had profound regrets about their own situation, especially if they cared for a parent rather than a spouse. Some studies also note the burdensome and poorly supported environment in which carers make decisions [33, 34, 39], both in dementia and traumatic brain injury. Others highlight the feelings of guilt or failure family carers of people with dementia bear [25], especially about decisions that violate relatives’ perceived wishes and the strong sense of obligation carers may feel [35]. Relatives of people with dementia may feel particularly unsupported: one epidemiological study of end-of-life shows that presence of dementia in a dying relative is associated with family dissatisfaction with medical communication [40]. Attention to family wellbeing may vary according to the setting, with one study noting that families’ emotional needs were more likely to be recorded in hospital and hospice documentation than in nursing home records [41].

A sense of obligation may lead family members to wish for more treatment for their relatives than they would choose for themselves [42]. However, family members of people with dementia who participated in focus groups showed increased levels of regret if they had requested aggressive interventions at the end-of-life [25]. Such discordant thinking is also seen in health care professionals’ wishes for their patients [43, 44].

#### Weight of family views

Where families requested treatment in contradiction of previously agreed care plans [24, 34], many studies indicated that their wishes were likely to be adhered to, even if this was in opposition to the earlier expressed wishes of the relative [24, 33, 38, 45] (note there was no evidence that families *rejection* of treatment carried a similar weight). While this key role for the family was widespread in dementia, traumatic brain injury and frailty, one study indicated that family members were rarely consulted [36] and others felt they had little influence on decision-making [29]. This finding presumably reflects the broad scope of our literature search – one study highlights regional and international differences in medical values [43] – but there are also signs in the literature indicating that there can be dynamic differences in the manifestation of shared decision-making from one case to another.

#### Ambiguity of “shared decision-making”

It was implicit across studies of frailty [46, 47], dementia [25, 32, 35, 48–53] and traumatic brain injury [33, 54] that decisions were shared between doctors and families and/or patients. Moreover, regardless of clinical setting, doctors changing their treatment plans in response to family intervention may indicate that decision-making is to an extent shared [21, 24, 28, 55]. Yet this understanding of equitable authority is muddied by reports that family wishes were circumvented when they strayed from the (previously agreed) medical plan in palliative care settings [26] or where their view of what the patient might want was in opposition to a clinician’s assessment of best interests. In acute settings, while families often readily acceded to aggressive lifesaving treatments, families who opposed these were overruled by the healthcare team [34]. The concept of shared decision-making appears ambiguous, an ambiguity which is captured in the finding from a survey of family members of patients with traumatic brain injury that a majority of families felt their role was simultaneously to make decisions and to accede to decisions made by the doctor [33]. This sense of a blurring of the boundaries between making and agreeing to decisions is further compounded by one case study series, again of traumatic brain injury, which emphasises that decisions to withdraw nutrition and hydration were made by doctors because of their challenging nature [56].

#### The role of incapacitated patients in decisions

An important aspect of decision-making is the role that legally incapacitated patients may play. This role is easily overlooked, first because the patient is not considered to have capacity to make decisions, and secondly because many patients with high levels of dependency display overt unwillingness to engage with decision-making. One survey indicated that many people with dementia wished others, usually trusted family members, to make decisions on their behalf [57]. Further, many people with dementia and frailty in old age did not wish to discuss end-of-life issues [21, 45] and people in the middle stages of dementia could resist change and deny problems to their families and family doctors (General Practitioners, hereafter GPs), especially if they were used to holding an authoritative role in the family [25]; in these cases families and GPs often put on a united front to overcome this resistance.

While a lack of formal capacity results in routine marginalisation [28] or even exclusion from decision-making [24, 49, 53, 58] of frail older people and people with dementia, the documented resistance of people with dementia and/or frailty in old age indicates an ability to influence decisions. The difficulties of engaging in discussion with a previously authoritative parent or relative [25] indicate that family dynamics may exert pressure on how decisions happen. Indeed, this speaks to findings that the identity of carers can have a measurable impact on decision-making: an epidemiological study of decisions to forego hospitalisation in advanced dementia [59] showed a strong association between patients having a proxy who was not a child and possessing a ‘do not hospitalise’ order, which the researchers suggest indicates that patients’ children may be less willing to limit treatment than other relations (including spouses) or court appointed proxies. Similarly, a survey by Rurup et al. [31] notes that, while many family members agreed with limitations to treatment for relatives at the end-of-life, children and spouses agreed less often than those with other familial relationships.

A variety of influences can be glimpsed in the literature; the fact that individuals with traumatic brain injury survive at all, ‘against the odds’, in intensive care units can be seen as grounds for hope by families [22, 33, 34]. This is significant given that Jox et al. [33] note that the family’s perception of the patient’s wellbeing is more frequently influential than the patient’s advance directives or past attitudes. Clearly there is a symbolic role that signs and symptoms of illness play in family reasoning. Yet overt influences on decision-making that rely on the patient’s physical and verbal behaviours are also glimpsed in the literature. One survey of attitudes to artificial nutrition and hydration noted that when nursing home residents with advanced dementia refused to eat and drink, a large minority of relatives and nurses (although fewer doctors) agreed that this refusal should be ‘respected’ [31]; another study noted that GPs’ decisions to hospitalise frail older persons who lacked capacity to make decisions were influenced by the patient’s verbal wishes as expressed at the time [55]. Such observations offer a glimpse at the various ways that people with impaired decision-making ability influence the decisions of others, and such influence is recognised in law in some jurisdictions,^3^ the paucity of sources in this review may suggest that this is an under-researched area in this literature.

### Theme 2: limiting the intensity of medical treatment

While only one study explicitly examined the issue of a ‘good death’, many other studies took an implicit position on a good death, viewing this as a death that was not overly medicalised. The aetiology of illness dictates the type of medicalisation to be avoided, and in the conditions under investigation, medical omissions were often of life-saving drugs (commonly antibiotics) and of artificial nutrition and hydration (usually pertaining to liquid feeds that were administered via nasogastric or gastrostomy tube). While avoiding hospital or aggressive treatment was associated with the idea of a good death, many patients and carers expressed an active wish for medical intervention, especially if confronted with acute deterioration or imminent death. Thus there was a substantial paradox where not only a natural death, but also aggressive life-saving treatments, were desired. Indeed, misconceptions of the type of death a relative could expect may be compounded by under-recognition of the trajectory of degenerative conditions among medical professionals, affecting the information they communicate to families.

#### Patterns of medicalisation

A number of studies documented the degree of medicalisation of deaths, with one study reporting that almost 1 in 5 patients received ventilation, resuscitation or surgery in the last 48 h of life [41]. Di Giulio et al. [36] observed the frequent use of restraint, antibiotics and feeding by gastric tube at hospital institutions caring for those dying with advanced dementia, and others reported a comparative reduction in these measures in nursing homes and residential care [40]. While studies reported wide variations between hospitalisation rates from different nursing homes [60, 61], Lamberg et al. [59] reviewed the records of 244 nursing home residents dying with advanced dementia, and noted that about 20 % had a period of hospitalisation in the last six months of life. Similarly, relatively high proportions of frail older nursing home residents had contact with out-of-hours care and ambulance services in the month prior to their deaths [21]. When compared to other types of long-term patients, length of hospital stay of people with dementia and cognitive impairment were lower [40, 61, 62]. Basic and Shanley [63] reported that people with dementia were less likely to die during hospital admission, speculating this may be because those who are hypermobile have frequent review by their doctors and low nurse-to-patient ratios. Basic and Shanley’s findings are tempered somewhat by the findings of a cohort study of dementia patients by Sampson et al. [64] which reported that the likelihood of mortality for dementia patient’s in acute hospitals was nevertheless high, and increased as cognitive impairment or dementia worsened. I am grateful to a peer reviewer for this information.

#### Appropriate medicalisation

It is noteworthy that some studies disputed the development of a dogmatic approach to de-medicalised death. For instance, while some studies indicate that vexatious factors such as overwork, poor communication and potential for litigation played a part in clinical decisions to hospitalise nursing home patients [24], other authors argue that some clinical emergencies, such as electrolyte imbalances and fractures, would be inappropriate to keep out of hospital [55], and similar themes were highlighted by relatives of older home hospice patients who requested hospital in the final days of life [26]. Patients may not equate a de-medicalised death with a good death; as Soskis [65] notes, patients from marginalised communities “are far more likely to worry that treatment providers will give up on them too soon or will not consider them worth saving”.

#### Predicting death

Given the common steer toward a death that was not unnecessarily medicalised, a major impediment to avoiding prolongation of life for its own sake is distinguishing terminal trajectories from other life threatening emergencies [21, 38, 66]. Lamberg et al. [59] note that almost half of decisions not to hospitalise nursing home residents with dementia took place during the last 30 days of life, while more than half of decisions to palliate rather than actively treat took place in the last 7 days of life. The relative lateness of such planning may of course be related to a lack of recognition that dementia is a cause of death in its own right, highlighted in the previous theme. Such a conclusion is in broad agreement with a finding that GPs more frequently recognise when deaths from cancer were about to take place than deaths from all other conditions [46]. These authors note that where death is not anticipated, sudden deterioration is more likely to result in hospitalisation rather than palliative care.

#### What constitutes a ‘good death’?

What does, and does not, constitute a ‘good death’ is a major area of academic debate, and this was not directly examined in the scoping review. As noted above, many studies took an implicit position on the meaning of a ‘good death’ by problematising the degree to which death is medicalised. Only one study, by Bosek et al. [30], engaged with the literature on a good death explicitly, attempting to reconcile family members’ perspectives on the quality of death of a recently deceased relative with late-stage Alzheimer’s disease with the Steinhauser et al. [67] typology of good death. Bosek et al. [30] report that, while many families were not at the bedside when their relative died, most felt that their relative had died with dignity. This notwithstanding, more than a quarter of family members felt their relative had not experienced a good death. Impediments to a good death included undesired symptoms and, in more than half of cases, failure to fulfil the antecedent wishes of the individual. Bosek et al. [30] link this to the concepts of symptom control and planned death that are central to the Steinhauser et al. [67] typology, concluding that the cognitive impairments late stage dementia present severe challenges to achieving a good death in Steinhauser’s terms, and calling for a more proactive approach to end of life care.

#### Shifting perceptions of dying well

Effective symptom control may be important, but understanding what constitutes a symptom requires a particular reference point, and some studies identify the unpreparedness of family members for the dying process. For instance, a study of nurses’ and relatives’ experiences of continuous deep sedation of dying dementia patients theorised that differences in the familiarity of relatives and nurses with the dying process led to differences in their perceptions of dying as (for instance) peaceful or agonising [32]. Several other studies had similar findings; thus studies observed antecedent wishes expressed by the patient to avoid machines or extraordinary measures [68] or the frequent desire for natural death among family members [35], but noted the lack of specific information about what such deaths would involve. Indeed, families may offer paradoxical visions of good death; thus, while electing not to treat pneumonia and other infectious diseases is sometimes seen as allowing a natural death, both Forbes et al. [35] and Potkins et al. [48] note that failing to treat such conditions was often not acceptable to members of many families of people with dementia (although, where dementia is severe, there may be a rise in acceptability). It is notable that death does not always follow withholding and withdrawing antibiotics or other treatments [34, 56]. Regardless of this, a study of home hospice patients indicates that the reality of the symptoms and sequelae of the dying process prompts some families to request hospitalisation in the final stages of life [26]. There is also a consistent tendency among both professionals and family members toward requesting more clinical interventions for others than they would wish for themselves [42, 44].

Requests for medical interventions are also made by families of patients in prolonged or permanent vegetative states [33, 34]. While often thinking their relative has an extremely poor quality of life, many families strongly resist withdrawal of artificial nutrition and hydration [22, 33], despite this being a legally permissible avenue to a ‘natural death’ for this condition in some jurisdictions. Clinicians were more likely than families to find withdrawal of artificial nutrition and hydration acceptable [37, 43, 56].

### Theme 3: antecedent end-of-life decision-making

The final theme was concerned with informing future end-of-life treatment in advance. A bewildering range of terms—some legal, some bespoke—for such future planning were used, and the term antecedent end-of-life decision-making (AEDM) is used here to include all the terms encountered. Despite evidence of international momentum in adopting AEDM practices, there is apparent reluctance among some target populations to engage in antecedent end-of-life decision-making and still large number of obstacles confronting those who do want to do so, and the literature described a range of barriers patients, families and professionals encounter when trying to act in accordance with antecedent wishes. The literature revealed that families and health care professionals find discussing death difficult, and that documentary forms of AEDM (see below) are often felt to have little influence on decisions in clinical practice. Implicit in many studies was a wide variety of attitudes to death and dying, which may speak against a single approach to end-of-life decision-making. Despite this, some studies succeeded in promoting antecedent end-of-life decision-making, and documented its benefits.

#### A multitude of terms

A great many terms were used to describe AEDM within the papers. In part, this was due to the diversity of this review, drawing on papers from 13 different countries. In total the studies used 16 terms: advance directive, written advance directive, advance decision, advance care plan, documented advance care wishes, anticipatory care plan, power of attorney, durable power of attorney, durable power of attorney for healthcare, living will, values history, previously expressed wishes, previously expressed patient wishes, family physician treatment order, resident and family wishes, and familial advanced plan. Some of these terms are legally defined, some are not, (or are inaccurate attempts to reference legal terms) and the latter may be unique to the studies within which they are used. It appears evident that there are areas of overlap between these terms, although there are also important differences; however, exploring these differences, or the legal frameworks of different jurisdictions, is not the focus this paper.

The most common terms were advance directives and advance care plan, understood (in these studies and more generally) to represent respectively legally binding refusals of treatment, and non-legally binding statements of wishes and preferences. The UK uses the term advance decision, rather than advance directive, which is preferred in the USA (and refers to a broader range of advance planning instruments). To avoid ambiguity, we use only the terms *advance decision* and *advance care plan* in this discussion, or where both are implied, *antecedent end-of-life decision-making*.^4^

The abundance of different terminology used in the literature may give rise to confusion among health care professionals, and one UK study indicated that professionals were unsure of the difference between an advance decision (the legally binding refusal of treatment) and the more generic ‘advance care plan’ [45].

#### Support for antecedent end-of-life decision-making

When the period preceding death is characterised by a loss of capacity, carers and clinicians face ethical dilemmas about the types of treatment they should instigate or withhold. The papers reviewed are unanimous in considering end-of-life care based upon the patient’s antecedent wishes to be the gold standard in end-of-life treatment. While this is in line with the mores of western medicine, this review indicates that this is increasingly an international viewpoint, which is reflected in research produced in countries with little social history of such interventions [47, 69] - albeit these studies take place in Hong-Kong which is likely to be more susceptible to the influence of British practice than mainland China.

#### Barriers to antecedent end-of-life decision-making

Antecedent end-of life decision-making is a potential solution to the ethical dilemmas faced by clinicians and carers, yet this method of decision-making has failed to permeate practice. In the USA, where advance decisions to refuse treatment have been legally enforceable since the early 1990s and has received extensive publicity, indications are that only a third of adults have completed some form of antecedent decision [70]. In the UK, where advance decisions were given legal force in 2005, although with little publicity, only 5 % of people are reported to have formally engaged in AEDM – with fewer taking advantage of the legal instruments [71].

The papers in this review bear out these figures, indicating that some patients do not want to engage in antecedent end of life decision-making, or face obstacles to doing so. People with dementia [28, 45] and frail older people [21, 47], may be reluctant to think about their deaths and consider future end-of-life wishes. This reluctance is also felt by some members of families of people with dementia [25, 39, 68] and is echoed in the responses of some families of patients with severe brain injury who may resist making decisions to limit medical treatment even when injuries are catastrophic [34].

Dementia studies indicate that healthcare professionals can find it difficult to engage in discussions about end-of-life wishes with patients [35, 49]. While other studies report difficulties in engaging patients with advance care planning irrespective of primary diagnosis [21, 27, 45], the symptoms of dementia may themselves cause problems with advance care planning. Dening et al. [28] note that the nature of the impairments people with dementia experience means that they are more likely to focus on their immediate concerns rather than on the future, and this may present an obstacle to AEDM for those in the early stages of the disease. Reluctance to discuss end-of-life wishes can be exacerbated by a lack of close relationships between staff and families, which studies reveal can be a particular problem in nursing homes due to high staff turnover [35, 38].

#### Variations among individuals and families

Families are not always uncomfortable talking about death and making appropriate plans [34], especially if they have personally witnessed situations where someone has experienced a ‘bad’ death [25, 28]. These and other studies demonstrate clear variations in attitudes toward death and dying among different clinicians [37, 58], families [48, 72] and patients [65], but few show these variations in values as clearly as Chan and Pang [47], whose study offers a typology of nursing home residents’ approaches to advance care planning. While having different end-of-life preferences, residents also varied in their decisiveness, readiness to think about their deaths, and degree of fatalism. It is clear that strategies could be developed to address some of these barriers, yet the study indicates that any homogenous approach to AEDM is likely to encounter significant numbers of patients and/or families to whom it is personally ill-suited.

#### The efficacy of advance care planning

Advance care plans (excluding advance decisions) have an advisory, rather than determinative, effect on decision-making and dementia studies indicated that they are overridden in the case of strong opposition from families [35, 49]. As noted earlier, family members’ perceptions of their relative’s wellbeing had more influence on their decisions than the relative’s oral advance wishes and past attitudes [35, 38], and the contemporary behaviours of incapacitated patients also influenced decision-making. There was little faith in the utility of advance care plans among some patients, families [28] and clinicians [38]. Indeed, the difficulties of fitting the precepts of advance care planning to situations that involve illness trajectories other than cancer has been questioned by dementia practitioners drawn from a variety of settings [45].

#### Overriding advance decisions

Both dementia and frailty studies report advance decisions being overridden where there had been an antecedent rejection of artificial nutrition and hydration [23] and hospitalisation [59, 61].^5^ A survey by Rurup et al. [31] found that many more relatives and nurses than doctors felt that advance decisions should always be respected; despite this, relatives’ satisfaction with end-of-life care was more heavily influenced by a positive, trusting relationship with the treating clinician than compliance with the relative’s prior wishes [26].

Even where they are respected, both advance decisions and advance care plans vary greatly in determinative quality and content. While some studies indicate moderate agreement between surrogate reports and documented patient wishes, the instructions may be non-specific, fail to address common end-of-life dilemmas (e.g. tube feeding and hospitalisation), or contain contradictory wishes or illegal requests (e.g. for euthanasia in the U.K.) [25, 50, 57, 65, 68].

#### Promoting advance care plans and advance decisions

A number of the included intervention studies were carried out within care settings for older people with dementia or frailty. Interventions such as staff or family education and facilitation encouraged families and patients to document antecedent end-of-life decisions [27, 39, 47, 52, 65, 66]. Many of these studies suggest that benefits arise from antecedent end-of-life decision-making. Frail older people who were involved in advance care planning showed more life satisfaction, less existential distress and increased preference stability [47], while GPs reported reductions in care costs and hospital admissions [66]. Interventions caused bereaved families of persons with dementia to report increased satisfaction with end-of-life care and increased numbers of documented advance care plans [39] and advance decisions [52] in care homes. These findings notwithstanding, a study in an acute hospital setting reported that relatives were frequently unwilling to make advance care plans for family members with dementia, even with expert guidance, and concluded that relatives may be ill-suited to the task in these circumstances [27]. Thus, this limited range of literature suggests the success of interventions to promote AEDM may depend on a variety of factors, including the setting and the participants, and this deserves further investigation.

## Discussion

The three themes show that clear links can be made between the three groups of patients with reduced agency. The first theme concerns the processes of end-of-life decision-making, highlighting the ambiguity of the dominant shared decision-making process, wherein decisions are determined by families or doctors, sometimes explicitly marginalising the current or antecedent decisions of patients. Despite this marginalisation, the patient played a role both as a social presence whose past identity and present clinical signs could be influential, and as an active agent influencing the decisions of those with authority. The second theme examines the tension between predominant notions of a good death as ‘natural’ and the drive to medicalise death through the experiences and actions of families faced with the actuality of death, and the impulse to treat among healthcare professions. The final theme considers the concept of antecedent end-of-life decision-making (in all its forms), its influence on policy and decision-making and some caveats that arise from the studies.

### Shared decision-making: family consultation, the power of doctors and the exclusion of patient agency

Not unsurprisingly, given the legal, ethical and cultural weight afforded to families’ views, the review indicated that the families are the default proxies in a process of shared decision-making. However, the importance given to families’ opinions, rather than doctors’ opinions, varied among the studies. Whatever the relative influence of these parties, reports of advance care plans being overridden by families or advance decisions being overruled by doctors indicate the marginalisation of the wishes of the patients themselves. This also highlights the ambiguous nature of the concept of ‘shared’ decision-making, given that any weighting of opinions short of complete exclusion could be argued to involve sharing. Indeed, given the power of doctors to make decisions in the best interests of their patients, shared decision-making appeared sometimes to reduce friction with, and lessen the burden on, families, rather than offering them a determinative role in decision-making.

The ambiguity of shared decision-making has been examined at length in the literature [20, 73, 74], and the contest between families and clinical staff over what is best for patients is familiar [75]. Yet this description hides a much more complex situation, glimpsed, but not directly examined, by the studies. The way agency is exerted—sometimes subversively—by older people who are deemed to lack the ability to formally make decisions has been noted by anthropologists, who document the rebellion against resented authority that manifests in soiling or refusing food [76]. Such a lens seems absent from the literature reviewed, but suggests that mentally incapacitated patients might more realistically be viewed as participants in the decision-making process, exerting influence in a multitude of ways. Such influences may have biographical roots – such as the difficulty in managing a previously authoritative parent – but also seem to illustrate how cues in behaviours (such as patients refusing food) or verbal wishes (demanding hospitalisation) play an active role in others’ decisions even where the patient is not considered to be of ‘sound’ mind. In the UK the law does go some way to acknowledging this influence, but its weight is not settled [77]. While seeking information from families is clearly necessary in many cases (including to ascertain applicable past wishes of patients who lack written statements), the views of proxies should be balanced with the past and – importantly – the present voices of incapacitated patients. Patient agency in end-of-life decision making should therefore form a focus of future inquiry.

### ‘The good death’ in theory and practice

Limiting the intensity of medical treatment was a major focus of studies and underlined the implicit position that a ‘good death’ is one that minimises medical intervention and maximises choice. Maximisation of choice seems to agree with broader perspectives on good death elsewhere in the literature, and also reflects a policy focus that has so far focused on death primarily in the context of cancer [4]. Yet there are questions about the relevance of these concepts to longer-term degenerative illness, where formal decision-making ability may decline or be lost all together.

Minimising medicalisation should also not be seen as a given: a number of studies underlined the fact that a death without medical intervention may not always be seen by patients (in advance care plans) or families (in studies of satisfaction) as a good death. The actual circumstances of death, where the differences between what is expected and what is possible markedly diverge, may cause families to reassess prior plans, and to disregard prior decisions agreed with the patient. These gaps between expectation and reality may be driven by poor understanding of how death happens, suggesting a need not simply or solely to furnish families with less sanitised understandings of dying, but also to remedy apparent failings of professional perceptions of death from degenerative conditions. Given the failings of current policy, correcting multiple layers of public, professional and policy-maker misunderstanding represents a major challenge, albeit one which future work could usefully seek to address.

### Antecedent end-of-life decision-making: one size fits all?

While the multitude of terms used in English language studies of antecedent decision-making might cause confusion among healthcare professionals, the increasingly international nature of antecedent end-of-life decision making is evidenced in this review, is perhaps most notable when considered beside the difficulties faced in engaging the public with antecedent end-of life decision-making in jurisdictions where it is well-established.

While it remains difficult to engage some families and patients, some studies have managed to overcome this reluctance and promote more widespread AEDM (although this experience is not universal). However, once such a decision is in place, it is apparent from other studies that it may not be respected – indeed, advisory instruments like advance care plans seem to be readily overridden in a process that speaks of the marginalisation of incapacitated patients, as discussed above. Besides these difficulties, the existence of a variety of different personal attitudes to decision-making suggests that a homogenous process offering only a binary in/out approach to AEDM may exclude large sections of the public.

Difficulties in communicating about death speak to the observation that dying is little understood, but even where death is accurately anticipated there were sometimes differences between current and prior wishes, and the effects of these temporal elements of decision-making seem to be unexamined in the literature pertaining to this theme. There may be important qualitative reasons why antecedent and contemporary decisions are denied equivalence that may be germane to questions about why many people are (apparently) unmoved by antecedent decision-making in this context. This, too, could be a fruitful area for future inquiry.

### Limitations

This study concentrated on scope rather than depth, and moreover explicitly imposed time constraints within the search methodology; thus it is more than likely that important studies have been overlooked and conclusions are highly provisional. Derivation of themes is a subjective process, even with such themes being checked by a second reviewer, and it is possible that others would offer different readings of key data. Moreover, the research question that guided the literature search, in seeking both differences and similarities between settings and groups may have been better answered by analytic techniques that accentuate difference and similarity. Thematic analysis is most potent in producing a broad sketch of multiple sources and may have therefore have under addressed the differences and similarities sought in the original research question. Nevertheless, given the novelty of our focus and the fact that many findings have arisen that are worthy of further research (which could include a more focused review), we believe that the aims of the review expressed at the outset of this paper have been met.

## Conclusions

This paper documents a scoping review and subsequent qualitative synthesis that finds common features in end-of-life care for a set of conditions that result in ‘reduced agency’. Three major themes arose from the review: First, the process of end-of-life decision-making took place in the context of the rhetoric of shared decision-making that included families and clinicians, but not, directly, the patients themselves. Patient wishes were sometimes overruled by doctors or families, which raises concerns about the fragility of the patient’s autonomy within this process. Nevertheless, patients lacking capacity influenced the decisions of others through a variety of passive and active means, and this dynamic is little studied in the papers reviewed here. Secondly, academic, public and professional discourse that presumed equivalence between minimal medical intervention in the dying process and a ‘good death’ vied with counterfactual requests and behaviours when faced with death in reality. Poor understanding of death may drive this paradox, not just among the public, but among policy makers and healthcare professionals, suggesting that corrective intervention to increase understanding is a major challenge. Finally, we noted the tension between the increasingly international adoption of antecedent end-of-life decision-making and the difficulties in progressing this approach in countries where it is well established. While containing some interventions to promote antecedent end-of-life decision-making, the studies also documented widespread barriers to their use. Not least of these was the variety of personal perspectives on death, which suggests that parallel approaches require development.

Together these three themes indicate a number of directions for future research in this area, which are likely to be broadly applicable to other conditions that result in reduced agency. Above all, this review emphasises the limits of current concepts and approaches to end-of-life decision-making, and the need for fresh approaches.

## Abbreviations

AEDM, antecedent end-of-life decision-making; DWRA, dying well with reduced agency; GPs, general practitioners; UK, United Kingdom; USA, United States of America

## Additional files

Additional file 1: Table S1. Summary of papers. (DOCX 20.8 kb) Additional file 2: Table S2. Theme development process. (DOCX 20.1 kb) Additional file 3: Table S3. Initial codes. (DOCX 18.6 kb)

## Footnotes

1 Other groups such as premature babies and people with learning disabilities are not considered here, as our focus is on adults who had legal capacity to make their own decisions, and have now lost it, or have severely reduced agency.

2 Individual searches were performed on these journals because we suspected they would publish papers particularly germane to our research question that a broad database search might overlook. Four papers, all from the *British Journal of General Practice* were included in the final review as a result.

3 Such an influence is recognised in UK statute and common law, although the degree of influence remains ambiguous e.g. *Re S and S (Protected Persons) Cases* 11475121 and 11475138 (COP) [2008]. We have not surveyed the legal position in other jurisdictions.

4 Additionally, UK law also includes the Lasting Power of Attorney for Health and Welfare where a person names a third party to make decisions about their health and welfare, including decisions to refuse treatment. In this review papers considered advance care planning and advance decisions, so this category of third party decision-making is not explicitly considered here.

5 These studies are of settings in the USA. For an outline of the US position on Advance Decisions see Rich [78].
