# Supplementary material for: Dying well with reduced agency: a scoping review and thematic synthesis of the decision-making process in dementia, traumatic brain injury and frailty
Source: BMC Med Ethics. 2016 Jul 27;17:46. doi: 10.1186/s12910-016-0129-x (PMC4962460; doi:10.1186/s12910-016-0129-x)
Supplement: Additional file 1: Table S1. — Summary of papers. (DOCX 20.8 kb) [file 12910_2016_129_MOESM1_ESM.docx]

| Author | Ref | Location | Study type | Participants | Site | Study aims |
| --- | --- | --- | --- | --- | --- | --- |
| Abarshi et al (2001) | [46] | Holland | Survey | 252 clinicians | Community and hospital | Do family doctors recognise approaching death |
| Allen et al (2003) | [72] | USA | Epidemiological cross sectional | 78 residents (using clinician / proxy observations) | Care Home | Identify nursing home residents who can participate in advance care planning |
| Anquinet et al (2013) | [32] | Belgium | Cross sectional survey | 11 decedents | Nursing home | Uses and decision-making process for deep sedation until death |
| Ayalon et al (2012) | [42] | Israel | Cross sectional interviews | 53 couples (total=106) | Psychogeriatric clinic | To evaluate concordance of patient / carer end of life preferences |
| Baker et al (2012) | [66] | Scotland | Interventional cohort study | 192 older people | Community | Is it possible to reduce hospitalisation though advance care planning? |
| Barclay et al (2014) | [21] | England | Qualitative interviews | Residents/clinicians about 121 decedents | Care home | Describe events and trajectory of deaths care homes |
| Basic and Shanley (2015) | [63] | Australia | Epidemiology | 2125 older people | Hospital | Does frailty predict mortality? |
| Black et al (2009) | [68] | USA | Qualitative interviews | 34 surrogate decision makers | Nursing home | How surrogates for people with dementia interpret patient end of life wishes |
| Bosek (2003) | [30] | USA | Qualitiative interviews | 57 family members | Nursing home | Evaluate patient experience of death and dying including decision-making |
| Bottrell et al (2001) | [24] | USA | Qualitative focus groups | 13 lead nurses | Nursing home | Why were nursing home patients hospitalised? |
| Brazil et al (2012) | [29] | Canada | Survey of families | Families of 111 decedents | Community | Family perceptions of patient centeredness of palliative care in last four weeks of life |
| Cavalieri et al (2002) | [49] | USA | Physician survey | 63 physicians | Private practice and hospital | Assess content and provision of advance care plans for people with Alzheimer’s disease |
| Chan and Pang (2010) | [47] | Hong Kong | Quasi-experimental | 121 older people | Care home | Willingness to engage in advance decision making when facilitation provided |
| Chan and Pang (2011) | [69] | Hong Kong | Qualitative interviews | 42 older people | Care home | Beliefs of older people about end of life decision making |
| Demertzi et al (2011) | [43] | Pan-European | Survey | 2475 conference delegates | Professional conference | Attitudes to minimally conscious state and vegetative state |
| Demertzi et al (2014) | [44] | Pan-European | Survey | 3332 conference delegates | Professional conference | Attitudes to locked-in syndrome |
| Dening et al (2012) | [38] | UK | Qualitative interviews | 50 staff and family bereaved by dementia | Mental health day centre | Identify barriers to quality end of life care for people with dementia |
| Dening et al (2013) | [28] | UK | Qualitative nominal group technique | 6 people with dementia; 5 carers; 6 carer / patient dyads | Memory service clinic | To explore process, generation and prioritisation of preferences for end-of-life care. |
| Di Giulio et al (2008) | [36] | Italy | Chart review | 141 people with dementia | Nursing home | Describe last month of life of people with severe dementia in clinical settings |
| Evans et al (2006) | [26] | USA | Qualitative interviews | 18 bereaved family members | Community / Hospital | Why did family caregivers request transfer to hospital from home hospice? |
| Forbes et al (2000) | [35] | USA | Qualitative focus groups | 28 family members | Care home | Describe familial end of life decision making process |
| Fried and Mor (1997) | [61] | USA | Epidemiology | 3782 older people | Care home | What are the predictors of hospitalisation in older people? |
| Haller and Gessert (2007) | [62] | USA | Epidemiology | 1494 decedents | Care home | Factors associated with aggressive intensive care at the end of life |
| Jox et al (2015) | [33] | Germany | Survey | 44 family members | Neurological rehabilitation unit | Family member attitudes and approaches to decision-making about patients in vegetative state |
| Kitzinger and Kitzinger (2013) | [34] | England | Qualitative interviews | 26 family members | Community | Family understanding of the time-sensitivity of withdrawal of treatment from patients in vegetative state |
| Kitzinger and Kitzinger (2015) | [22] | UK | Qualitative interviews | 51 family members | Community | Family attitudes to withdrawal of artificial nutrition and hydration from patients in vegetative and minimally conscious states |
| Lamberg et al (2005) | [59] | USA | Cohort demographics | 240 decedents | Nursing Home | What informed decision not to hospitalise nursing home residents dying with dementia |
| Lavrijsen et al (2005) | [56] | Holland | Case series review | Clinician of 5 patients in vegetative state | Care home | Describe treatment and withdrawal of patients in vegetative state |
| Livingston et al (2010) | [25] | UK | Qualitative interviews | 89 family members | Community | Barriers and facilitators to difficult decisions about dementia care |
| Livingston et al (2013) | [39] | UK | Qualitative intervention | 53 Proxies of 98 decedents | Care Home | Increase family satisfaction with end of life care |
| McDermott et al (2012) | [55] | England | Qualitative interviews | 21 clinicians | Community | What were the reasons for hospitalising of frail older people |
| Monteleoni and Clarke (2004) | [23] | USA | Intervention study | Clinicians of 40 patients | Hospital | Reduce feeding tube placement in advanced dementia |
| Mor et al (2005) | [60] | USA | Retrospective cohort study | 15460 decedents | Care home | Are there racial influences on degree of hospitalisation at end of life? |
| Nakanishi and Honda (2009) | [50] | Japan | Retrospective cross sectional survey | Home managers regarding 33 decedents | Group home | Content of end of life decisions for people with dementia |
| Parsons et al (2014) | [51] | Ireland / N Ireland | Factorial survey with vignettes | 1079 clinicians | Community / Hospital | Which medications do doctors stop or withhold prior to the end of life |
| Pijnenborg et al (1995) | [58] | Holland | Questionnaires and qualitative interviews | 405 clinicians | Community / Care home / Hospital | Why doctors withhold or withdraw treatment at the end of life? |
| Potkins et al (2000) | [48] | UK | Survey ; chart review | 50 carers | Hospital / Care home | What treatments do families agree to withhold prior to the end of life |
| Reinhardt et al (2014) | [52] | USA | Intervention study | 110 family members | Care home | Effect of structured conversation about end of care vs routine social contact on care planning |
| Robinson et al (2013) | [45] | UK | Qualitative interviews | 95 clinicians | Hospital / Care home / Community | Professional experiences of advance care planning for people with dementia |
| Rodrigue et al (2013) | [37] | Canada | Qualitative interviews | 21 clinicians | Hospital | Clinicians views about end of life decisions in coma and vegetative state |
| Rurup et al (2006) | [31] | Netherlands | Survey of clinicians and families | 421 clinicians and family members | Care home | Level of agreement of end of life decisions between Drs, Nurses and families |
| Sampson et al (2011) | [27] | UK | Action research (pilot study) | 33 carer/patient dyads | Hospital | Improve EOL care, encourage carers to write advance care plans |
| Sloane et al (2008) | [40] | USA | Cohort | 581 clinicians and 293 relatives of decedents | Nursing Home / Residential care | To understand unmet needs of people who die in long term care. Compare cognitively impaired vs intact, in care home vs in residential care. |
| Solloway et al (2005) | [41] | USA | Chart review | 782 decedents | Community / Care home / Hospital | Where did people die, what was their capacity and was an advance decision in place? |
| Soskis (1997) | [65] | USA | Qualitative intervention study | 23 older people | Care home | What is the content of advance directives? |
| Triplett et al (2008) | [57] | USA | Chart review | 123 residents | Nursing home | To examine how residents with advanced dementia conveyed wishes for care at the end of life. |
| Turgeon et al (2013) | [54] | Canada | Survey | 298 clinicians | Hospital | Clinicians’ attitudes and decision-making approaches in severe traumatic brain injury |
| van der Steen et al (2005) | [19] | Holland | Physician survey | 143 decedents reported by 86 physicians | Nursing home | Physicians intentions regarding hastened death at end of life when withholding antibiotics |
| Vandervoort et al (2014) | [53] | Belgium | Survey | Clinicians and relatives of 205 decedents | Nursing home | Examine awareness of end of life planning and congruence between dementia patients’ end of life decisions expressed to family and those documented by clinicians |
