# Supplementary material for: Dying well with reduced agency: a scoping review and thematic synthesis of the decision-making process in dementia, traumatic brain injury and frailty
Source: BMC Med Ethics. 2016 Jul 27;17:46. doi: 10.1186/s12910-016-0129-x (PMC4962460; doi:10.1186/s12910-016-0129-x)
Supplement: Additional file 2: Table S2. — Theme development process. (DOCX 20.1 kb) [file 12910_2016_129_MOESM2_ESM.docx]

| **Paper** | **Ref** | **Broad category 1:**  **Who informed the study?** | **Broad category 2:**  **Factors of end of life process examined** | **Theme 1**  **(Decision-making processes)** | **Theme 2**  **(Limiting treatment intensity)** | **Theme 3 (Antecedent end-of-life decision-making)** |
| --- | --- | --- | --- | --- | --- | --- |
| Abarshi et al (2001) | [46] | Clinicians | - Process of decision-making - Limiting treatment intensity - Antecedent end-of-life decision-making - Cost - Prognostication | - Ambiguity of "shared decision-making" | - Predicting death |  |
| Allen et al (2003) | [72] | Families and clinicians | - Antecedent end-of-life decision-making - Limiting treatment intensity | - Who decides? |  | - Variations among individuals and families |
| Anquinet et al (2013) | [32] | Families and clinicians | - Good death - Antecedent end-of-life decision-making - Nutrition - Setting | - Family and health care professional perspectives - Ambiguity of "shared decision-making" | - Shifting perceptions of dying well |  |
| Ayalon et al (2012) | [42] | Families | - Limiting treatment intensity - Process of decision-making - Nutrition | - Impact on families | - Shifting perceptions of dying well |  |
| Baker et al (2012) | [66] | Demographics | - Limiting treatment intensity - Antecedent end-of-life decision-making - Costs |  | - Predicting death | - Promoting advance care plans and advance decisions |
| Barclay et al (2014) | [21] | Families and clinicians | - Experience - Good death - Limiting treatment intensity - Process of decision-making - Antecedent end-of-life decision-making - Prognostication | - Who decides? - Ambiguity of "shared decision-making" - The role of incapacitated patients in decisions | - Patterns of medicalisation - Predicting death | - Barriers to antecedent end-of-life decision-making |
| Basic and Shanley (2015) | [63] | Demographics | - Prognostication |  | - Patterns of medicalisation |  |
| Black et al (2009) | [68] | Families | - Process of decision-making - Antecedent end-of-life decision-making - Good death |  | - Shifting perceptions of dying well | - Barriers to antecedent end-of-life decision-making - Overriding advance decisions |
| Bosek (2003) | [30] | Families | - Good death | - Family and health care professional perspectives | - What constitutes a 'good' death |  |
| Bottrell et al (2001) | [24] | Clinicians | - Limiting treatment intensity - Process of decision-making - Costs | - Who decides? - Weight of family views - Ambiguity of "shared decision-making" - The role of incapacitated patients in decisions | - Appropriate medicalisation |  |
| Brazil et al (2012) | [29] | Families | - Experience - Process of decision-making | - Who decides? - Weight of family views |  |  |
| Cavalieri et al (2002) | [49] | Clinicians | - Antecedent end-of-life decision-making | - Ambiguity of "shared decision-making" - The role of incapacitated patients in decisions |  | - Barriers to antecedent end-of-life decision-making - The efficacy of advance care planning |
| Chan and Pang (2010) | [47] | Patients | - Antecedent end-of-life decision-making | - Ambiguity of "shared decision-making" |  | - Barriers to antecedent end-of-life decision-making - Variations among individuals and families - Promoting advance care plans and advance decisions |
| Chan and Pang (2011) | [69] | Patients | - Good death - Antecedent end-of-life decision-making |  |  | - Support for antecedent end-of-life decision-making |
| Demertzi et al (2011) | [43] | Clinicians | - Nutrition - Good death | - Impact on families - Weight of family views | - Shifting perceptions of dying well |  |
| Demertzi et al (2014) | [44] | Clinicians | - Good death | - Impact on families | - Shifting perceptions of dying well |  |
| Dening et al (2012) | [38] | Families and clinicians | - Good death - Barriers (institutional) | - Impact on families - Weight of family views | - Predicting death | - Barriers to antecedent end-of-life decision-making - The efficacy of advance care planning |
| Dening et al (2013) | [28] | Families | - Antecedent end-of-life decision-making - Good death | - Who decides? - Ambiguity of "shared decision-making" - The role of incapacitated patients in decisions |  | - Barriers to antecedent end-of-life decision-making - Variations among individuals and families - The efficacy of advance care planning |
| Di Giulio et al (2008) | [36] | Demographics | - Drugs - Limiting treatment intensity - Good death - Prognostication | - Family and health care professional perspectives - Weight of family views | - Patterns of medicalisation |  |
| Evans et al (2006) | [26] | Families | - Limiting treatment intensity - Prognostication | - Who decides? - Ambiguity of "shared decision-making" | - Appropriate medicalisation - Shifting perceptions of dying well | - Overriding advance decisions |
| Forbes et al (2000) | [35] | Families | - Good death | - Family and health care professional perspectives - Impact on families - Ambiguity of "shared decision-making" | - Shifting perceptions of dying well | - Barriers to antecedent end-of-life decision-making - The efficacy of advance care planning |
| Fried and Mor (1997) | [61] | Demographics | - Limiting treatment intensity - Prognostication |  | - Patterns of medicalisation | - Overriding advance decisions |
| Haller and Gessert (2007) | [62] | Demographic | - Limiting treatment intensity - Cost |  | - Patterns of medicalisation |  |
| Jox et al (2015) | [33] | Families | - Process of decision-making - Antecedent end-of-life decision-making - Drugs - Nutrition - Prognostication | - Family and health care professional perspectives - Impact on families - Weight of family views - Ambiguity of "shared decision-making" - The role of incapacitated patients in decisions | - Shifting perceptions of dying well |  |
| Kitzinger and Kitzinger (2013) | [34] | Families | - Process of decision-making - Good death - Limiting treatment intensity - Drugs - Nutrition - Setting | - Family and health care professional perspectives - Impact on families - Weight of family views - Ambiguity of "shared decision-making" - The role of incapacitated patients in decisions | - Shifting perceptions of dying well | - Barriers to antecedent end-of-life decision-making - Variations among individuals and families |
| Kitzinger and Kitzinger (2015) | [22] | Families | - Nutrition - Good death - Limiting treatment intensity | - Who decides? - The role of incapacitated patients in decisions | - Shifting perceptions of dying well |  |
| Lamberg et al (2005) | [59] | Demographics | - Limiting treatment intensity - Good death | - The role of incapacitated patients in decisions | - Patterns of medicalisation - Predicting death | - Overriding advance decisions |
| Lavrijsen et al (2005) | [56] | Clinicians | - Process of decision-making - Nutrition | - Ambiguity of "shared decision-making" | - Shifting perceptions of dying well |  |
| Livingston et al (2010) | [25] | Families | - Good death - Experiences - Barriers (personal) | - Who decides? - Impact on families - Ambiguity of "shared decision-making" - The role of incapacitated patients in decisions |  | - Barriers to antecedent end-of-life decision-making - Variations among individuals and families - Overriding advance decisions |
| Livingston et al (2013) | [39] | Families and clinicians | - Antecedent end-of-life decision-making | - Impact on families |  | - Barriers to antecedent end-of-life decision-making - Promoting advance care plans and advance decisions |
| McDermott et al (2012) | [55] | Clinicians | - Limiting treatment intensity - Process of decision making | - Ambiguity of "shared decision-making" - The role of incapacitated patients in decisions | - Appropriate medicalisation |  |
| Monteleoni and Clarke (2004) | [23] | Demographics | - Limiting treatment intensity - Nutrition - Good death - Antecedent end-of-life decision-making | - Who decides? |  | - Overriding advance decisions |
| Mor et al (2005) | [60] | Demographics | - Limiting treatment intensity - Cost - Prognostication |  | - Patterns of medicalisation |  |
| Nakanishi and Honda (2009) | [50] | Clinicians | - Process of decision-making - Nutrition - Good death | - Ambiguity of "shared decision-making" |  | - Overriding advance decisions |
| Parsons et al (2014) | [51] | Clinicians | - Drugs - Limiting treatment intensity - Good death | - Ambiguity of "shared decision-making" |  |  |
| Pijnenborg et al (1995) | [58] | Clinicians | - Process of decision-making - Cost | - The role of incapacitated patients in decisions |  | - Variations among individuals and families |
| Potkins et al (2000) | [48] | Families | - Limiting treatment intensity - Nutrition - Drugs | - Ambiguity of "shared decision-making" | - Shifting perceptions of dying well | - Variations among individuals and families |
| Reinhardt et al (2014) | [52] | Families | - Good death - Antecedent end-of-life decision-making | - Ambiguity of "shared decision-making" |  | - Promoting advance care plans and advance decisions |
| Robinson et al (2013) | [45] | Clinicians | - Antecedent end-of-life decision-making - Barriers | - Weight of family views - The role of incapacitated patients in decisions |  | - Barriers to antecedent end-of-life decision-making - The efficacy of advance care planning |
| Rodrigue et al (2013) | [37] | Clinicians | - Process of decision-making - Nutrition - Prognostication | - Family and health care professional perspectives | - Shifting perceptions of dying well | - Variations among individuals and families |
| Rurup et al (2006) | [31] | Families and clinicians | - Nutrition - Process of decision-making | - Family and health care professional perspectives - The role of incapacitated patients in decisions |  | - Overriding advance decisions |
| Sampson et al (2011) | [27] | Families | - Antecedent end-of-life decision-making - Barriers | - Who decides? - Family and health care professional perspectives |  | - Barriers to antecedent end-of-life decision-making - Promoting advance care plans and advance decisions |
| Sloane et al (2008) | [40] | Families and clinicians | - Good death - Setting | - Impact on families | - Patterns of medicalisation |  |
| Solloway et al (2005) | [41] | Demographics | - Antecedent end-of-life decision-making - Setting | - Impact on families | - Patterns of medicalisation |  |
| Soskis (1997) | [65] | Patients | - Good death - Antecedent end-of-life decision-making |  | - Appropriate medicalisation | - Variations among individuals and families - Overriding advance decisions - Promoting advance care plans and advance decisions |
| Triplett et al (2008) | [57] | Patients | - Antecedent end-of-life decision-making - Good death | - The role of incapacitated patients in decisions |  | - Overriding advance decisions |
| Turgeon et al (2013) | [54] | Clinicians | - Process of decision-making - Prognostication | - Ambiguity of "shared decision-making" |  |  |
| van der Steen et al (2005) | [19] | Clinicians | - Drugs - Good death |  |  |  |
| Vandervoort et al (2014) | [53] | Families and clinicians | - Antecedent end-of-life decision-making - Process of decision-making | - Ambiguity of "shared decision-making" - The role of incapacitated patients in decisions |  |  |
