# Supplementary material for: Dying well with reduced agency: a scoping review and thematic synthesis of the decision-making process in dementia, traumatic brain injury and frailty
Source: BMC Med Ethics. 2016 Jul 27;17:46. doi: 10.1186/s12910-016-0129-x (PMC4962460; doi:10.1186/s12910-016-0129-x)
Supplement: Additional file 3: Table S3. — Initial codes. (DOCX 18.6 kb) [file 12910_2016_129_MOESM3_ESM.docx]

| Initial codes | |
| --- | --- |
| Broad category 1:  Who informed the study? | **Broad category 2:**  **Factors of end of life process examined** |
| Families These papers focus on the family. Families may be broadly defined here to include spouses, friends, surrogates and/or primary carers. Papers directly measure or consult families, rather than make assessments of families through others. Papers may be focused on how family members made decisions, either alone or on behalf of patients, how families interact with patients, family experiences, understandings or characteristics. They may (but will not necessarily) make assessments of patients, but (except in two anomalous cases; [27] where a patient focus-type group was consulted, [26] where a dyad of patient-carer consulted) these will be in terms of, or through interactions with, the family. | **Antecedent end-of-life decision-making** This theme was concerned with the way that advance decisions/directives, advance care plans or advance wishes could be implemented in practice, as well as the things that prevented their implementation. Papers included studies where the types of advance decisions residents or families wanted were investigated, the attitudes of families and professionals to advance decisions or advance care planning, end-of-life decision-making. |
| Families and clinicians In these papers the focus was shared between families and clinical staff (and sometimes other professionals). These papers asked both clinicians and families about their experiences or decisions, in parallel processes. | **Barriers** This theme identifies systemic problems with care implementation and delivery that prevented or reduced the efficacy of interventions. It has a wider scope than *Antecedent end-of-life decision-making*, to which it is related. |
| Clinicians These papers focus on the role and opinions of the doctors, nurses, care assistants or other professionals (usually, but not exclusively, those who planned and/or delivered care e.g. [43, 42]). They directly consult (e.g. in surveys, interviews) with these professional groups to understand practice. They may also consult with professionals to discover family opinions or behaviours, but do not directly engage with families. | **Costs** This theme relates to costs both as they relate to the monetary value of treatment and the use of scarce resources such as hospital beds, staff or equipment. It only includes attempts to limit hospitalisation if it is explicitly linked to resource use. |
| Demographics These papers use epidemiological methods to focus on demographic information about the target group that is gathered by instrumental measures. They may seek to understand decision-making or the experience of dying, but only via instrumental data (in other words, use of interviews or surveys excludes from this category). | **Drugs** This theme looks at drug use in end of life care, and/or attitudes toward their use. This includes the use of inotropes, antibiotics and statins, routine drugs to slow the progress of dementia and medications to relieve pain or anxiety or shorten life. |
| Patients These papers focus only on the patient/resident. They do this by either directly engaging with the patient (rather than mediating through the family) and/or examining the content of advanced decisions in decision-making. | **Good death** This theme is concerned with what a good death was or should be. While some explicitly identified factors in a good death, other evaluated the types of death that were experienced in certain situations without attaching a label of good or bad, but nevertheless communicating that improvement was possible, what end of life experiences were (un)acceptable or of good or poor quality etc. NB note large body of literature around this topic that we will not cover. |
|  | **Limiting treatment intensity** This theme specifically looks at the intensity of treatments at the end of life and in some cases pilot interventions to limit these treatments. Intensity includes hospital admissions, resuscitation, artificial nutrition and hydration and drug treatments. |
|  | **Nutrition** This theme examines the topic of artificial nutrition and hydration (ANH), both in the context of treatment withdrawal and treatment limitation. It includes information about acceptability of ANH use and withdrawal to various stakeholders. |
|  | **Process of decision-making** This theme offer reflections germane to the method by which decisions are made. This includes the way that families and doctors make decisions (or in some cases absent themselves from decisions) i.e. are decisions timely, who discusses decisions, when and where are decisions made, but excludes the values that drive end of life decision-making (these are covered by the theme *Good death*). |
|  | **Prognostication** This theme covers attempts to prognosticate and predict the course of care, severity of illness or the imminence of death, levels of hope or certainty and examinations of where predictable death is missed. |
|  | **Setting** This theme drew specific conclusions about the influence of setting on the type of end of life care that a patient received, e.g. abandonment by professionals. |
